# Construction of a gene knockout CHO cell line using a simple gene targeting method

**DOI:** 10.1186/1753-6561-9-S9-P2

**Published:** 2015-12-14

**Authors:** Marina Aga, Noriko Yamano, Toshitaka Kumamoto, Jana Frank, Masayoshi Onitsuka, Takeshi Omasa

**Affiliations:** 1 Graduate School of Advanced Technology and Science, Tokushima University, Tokushima, 770-8506, Japan; 2 Institute of Technology and Science, Tokushima University, Tokushima, 770-8506, Japan; 3 Graduate School of Engineering, Osaka University, Osaka, 565-0871, Japan

## Background

Therapeutic antibodies have become an important focus of the biopharmaceutical industry. The Chinese hamster ovary (CHO) cell line is a major host for therapeutic antibody production. To construct productive CHO cell lines, two major transfection methods are commonly used, i.e., random integration and gene targeting. Random integration is a common method in which randomly integrated transgenes cause variation in antibody productivity because they are located in various chromosomal regions that affect transgene expression levels. Recently, gene-targeting methods, in which exogenous genes are inserted into a specific chromosomal region, have improved remarkably. Gene targeting is based on homologous recombination using sequences targeting a specific genomic region of the host cell. Homologous sequences located on both sides of the exogenous gene are used. We used the recently developed clustered regularly interspaced short palindromic repeats (CRISPR)/CRISPR-associated proteins (Cas9) system as a gene-targeting method. The CRISPR-Cas9 system induces double-strand breaks (DSBs) via guide RNA and Cas9, which increases the efficiency of homologous recombination [1]. Guide RNA hybridizes to a target integration site and induces Cas9 protein expression, leading to DSB. Finally, the Cas9 protein cuts genomic DNA. In this study, we constructed a simple gene-targeting method in CHO cells using the CRISPR-Cas9 system in which CRISPR vectors induce DSBs and gene-targeting vectors are inserted at the DSB site. In the conventional method, gene-targeting vectors should contain homology arms for effective recombination. In this study, we used the CRISPER system without homology arms for gene-targeted recombination.

## Materials and methods

We constructed a CRISPR-Cas9 vector that expresses a guide RNA sequence targeting a region on chromosome O. Chromosome O was selected based on a previous classification of gene-amplified CHO cell chromosomes in order of decreasing size and assigned letters from A to T by fluorescence in situ hybridization (FISH) [2]. The CRISPR targeting sequence was determined from the BAC clone Cg0031N14, which contained the chromosome O sequence. Gene targeting vectors (pcDNA-GFP-DHFR) with or without target site homology arms were constructed from BAC clone Cg0031N14. The percentage of exogenous gene integration into chromosome O was determined by a FISH analysis. Total RNA extracted from E14Tg2a (mouse ES cells) was kindly provided by Dr. Tohru Kimura, Kitasato University, Kanagawa, Japan.

## Results

**Figure 1:**
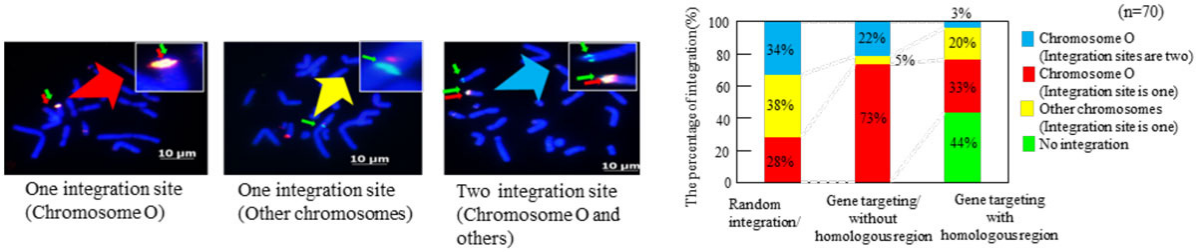
**The integration site and percentage of gene-targeting vectors using fluorescence *in situ *hybridization (FISH) analysis**.

To investigate the efficiency of CRISPR-Cas9 gene targeting in CHO cells, the frequency of gene integration into chromosome O was analyzed. In a previous study, chromosome O was identified as a suitable target site for stable and highly efficient exogenous gene expression [3]. In Figure 1, red signals indicate the BAC clone Cg0031N14 hybridized to chromosome O. Green signals show the integration sites of gene-targeting vectors (Figure 1). Using the random integration method, 29% of transfected cells showed specific integration into chromosome O and 71% of cells showed integration into other chromosomes (Figure 1). For integration using the antibody vectors with homology arms, 33% of transfected cells showed specific integration into chromosome O, 23% of the cells showed integration into other chromosomes, and 44% of the cells were not observed (Figure 1). Using the antibody vectors without homology arms, 74% of cells showed specific integration into chromosome O and 26% of cells showed integration into other chromosomes (Figure 1). These results indicated that exogenous genes can be efficiently inserted into a specific region of the genome (e.g., chromosome O) using CRISPR-Cas9 vectors, without adding homologous regions to both sides of the exogenous gene

Using the CRISPR-Cas9 system without homologous regions, we performed knockouts of de novo DNA methyltransferase genes in CHO cells. The cellular productivity of a gene-of-interest (GOI) is known to decrease during long-term cultivation. DNA methylation is closely related to this decrease in productivity. We constructed methyltransferase-knockout CHO cells for stable production. The expression levels of the de novo DNA methyltransferases Dnmt3a, 3b, and 3L in CHO cells have not been examined previously. We investigated the expression levels of these methyltransferases using RT-PCR. E14Tg2a cells (mouse ES cells) were used as a positive control and express Dnmt3a, 3b, and 3L. Only Dnmt3a was expressed in CHO-K1 cells, while Dnmt3b and 3L were not detected. Therefore, we focused on the downregulation of Dnmt3a expression in CHO cells using the CRISPR-Cas9 system without homologous regions that we developed. The CRISPR-targeting sequence was determined based on the Dnmt3a activation site in exon 19 and Dnmt3a expression was knocked out (Target 1). The CRISPR vector was constructed 5 bases from the Dnmt3a stop codon as the second target (Target 2). For Targets 1 and 2, Dnmt3a knock-out CHO cell lines were constructed using the CRISPR-Cas9 system without homologous regions.

## Conclusions

We were able to efficiently insert exogenous genes into a specific genomic region using the simple CRISPR-Cas9 vector system; additionally, Dnmt3a knock-out CHO cell lines were successfully constructed using this method.

## Acknowledgements

This work was partly funded by a grant for the Project focused on developing key technology of discovering and manufacturing drug for next-generation treatment and diagnosis from the Ministry of Economy, Trade and Industry of Japan and partly by a Grant-in-Aid for Scientific Research from the Japan Society for the Promotion of Science (JSPS) (No.26630433, 26249125). We are grateful to Dr. Tohru Kimura for providing total RNA extracted from E14Tg2a.
